# EMOTION WORD USE AND CARDIOVASCULAR REACTIVITY DURING MARITAL INTERACTIONS

**DOI:** 10.1093/geroni/igac059.733

**Published:** 2022-12-20

**Authors:** Tabea Meier, Jacquelyn Stephens, Claudia Haase

**Affiliations:** Northwestern University, Evanston, Illinois, United States; Northwestern University, Evanston, Illinois, United States; Northwestern University, Evanston, Illinois, United States

## Abstract

Cardiovascular reactivity in midlife may predict health problems in later life, but few studies have examined cardiovascular reactivity during marital interactions, as an important interpersonal context, and potentially modifiable linguistic correlates. This laboratory-based study examined emotion word use (i.e., positive and negative emotion words derived using automated language analysis) and cardiovascular reactivity (i.e., heart rate changes from baseline) across two marital interaction contexts (i.e., positive and conflict conversation) in 46 married couples (92 individuals; age: M = 42.6, SD = 8.5). Results showed that (1) spouses who used more negative emotion words during conflict showed higher cardiovascular reactivity. Moreover, (2) when husbands used a more diverse negative emotion word vocabulary during positive conversations, their wives showed higher cardiovascular reactivity and (3) when wives used a more diverse positive emotion vocabulary, their husbands showed lower cardiovascular reactivity. Findings highlight the relevance of couples’ emotion word use for cardiovascular reactivity in midlife.

